# Ectodomains of the LDL Receptor-Related Proteins LRP1b and LRP4 Have Anchorage Independent Functions *In Vivo*

**DOI:** 10.1371/journal.pone.0009960

**Published:** 2010-04-07

**Authors:** Martin F. Dietrich, Louise van der Weyden, Haydn M. Prosser, Allan Bradley, Joachim Herz, David J. Adams

**Affiliations:** 1 Department of Molecular Genetics, UT Southwestern, Dallas, Texas, United States of America; 2 Experimental Cancer Genetics, Wellcome Trust Sanger Institute, Hinxton, Cambs, United Kingdom; 3 Mouse Genomics, Wellcome Trust Sanger Institute, Hinxton, Cambs, United Kingdom; Leiden University Medical Center, Netherlands

## Abstract

**Background:**

The low-density lipoprotein (LDL) receptor gene family is a highly conserved group of membrane receptors with diverse functions in developmental processes, lipoprotein trafficking, and cell signaling. The low-density lipoprotein (LDL) receptor-related protein 1b (*LRP1B*) was reported to be deleted in several types of human malignancies, including non-small cell lung cancer. Our group has previously reported that a distal extracellular truncation of murine *Lrp1b* that is predicted to secrete the entire intact extracellular domain (ECD) is fully viable with no apparent phenotype.

**Methods and Principal Findings:**

Here, we have used a gene targeting approach to create two mouse lines carrying internally rearranged exons of *Lrp1b* that are predicted to truncate the protein closer to the N-terminus and to prevent normal trafficking through the secretary pathway. Both mutations result in early embryonic lethality, but, as expected from the restricted expression pattern of LRP1b *in vivo*, loss of *Lrp1b* does not cause cellular lethality as homozygous *Lrp1b*-deficient blastocysts can be propagated normally in culture. This is similar to findings for another LDL receptor family member, *Lrp4*. We provide *in vitro* evidence that Lrp4 undergoes regulated intramembraneous processing through metalloproteases and γ-secretase cleavage. We further demonstrate negative regulation of the Wnt signaling pathway by the soluble extracellular domain.

**Conclusions and Significance:**

Our results underline a crucial role for *Lrp1b* in development. The expression in mice of truncated alleles of *Lrp1b* and *Lrp4* with deletions of the transmembrane and intracellular domains leads to release of the extracellular domain into the extracellular space, which is sufficient to confer viability. In contrast, null mutations are embryonically (*Lrp1b*) or perinatally (*Lrp4*) lethal. These findings suggest that the extracellular domains of both proteins may function as a scavenger for signaling ligands or signal modulators in the extracellular space, thereby preserving signaling thresholds that are critical for embryonic development, as well as for the clear, but poorly understood role of *LRP1b* in cancer.

## Introduction

The LDL receptor gene family is a highly conserved class of cell surface receptors involved in various functions, including cell signaling, cargo transport, and gene regulation [1]. *LRP1b*, initially named *LRP-DIT* (Deleted in Tumors), was first described as a gene that was frequently inactivated in non-small cell lung cancer [2]. It was subsequently also shown to be mutated in urothelial [3], head and neck [4], [5], esophageal tumors [6] and in B-cell lymphomas [7]. The specific deletion of *LRP1b* in certain tumors through genetic and epigenetic silencing suggests a role as a tumor suppressor. However, the exact mechanism by which *LRP1b* functions in this manner remains elusive. *LRP1* and *LRP1b* share 86 percent mRNA and 52 percent amino acid identity. Previously reported mechanisms of action for *LRP1b*, including the regulation of the urokinase (uPAR) and platelet-derived growth factor (PDGF) receptor trafficking at the membrane level [8], [9], overlap with the functions of expressed and unmutated *LRP1* in tumor tissues. We have previously reported that mice that express of a truncated allele lacking both the transmembrane and intracellular domains of Lrp1b is viable [10].

Here, we extend our earlier findings by demonstrating embryonic lethality of two lines of mice carrying null alleles of *Lrp1b*. Interestingly, similar observations were made with the *Lrp4* knockout mice [11]. While *Lrp4* knockout mice fail to develop neuromuscular junctions and succumbed to respiratory failure post-natally [12], a truncated allele lacking the transmembrane and intracellular domains displays a mitigated phenotype compatible with postnatal survival [11], [13]. The common feature of the truncated *Lrp1b* and *Lrp4* alleles is that they secrete an intact and apparently physiologically functional extracellular domain.

All members of the LDL receptor gene family harbor at least one structurally highly similar extracellular ligand binding domain consisting of a series of negatively charged cysteine-rich Ca^2+^-chelating repeat modules that bind numerous ligands [14]. These ligand binding domains have numerous and partially overlapping functions in cell signaling and cargo transport [15]. The ability of the extracellular domain (ECD) to rescue embryonic or perinatal lethality suggests a functional role for the isolated ECDs.

We therefore propose a model in which the ECDs of LRP1b and LRP4 may modulate cellular signaling by scavenging and neutralizing extracellular ligands, thereby preserving signaling thresholds that are critical for proper embryonic development. The same mechanisms could impact on the development and progression of some malignancies.

## Materials and Methods

### Generation of Lrp1b-deficient mice

Mice carrying two different *Lrp1b* alleles were generated. These lines were termed *Lrp1b^tm1wtsi^* and *Lrp1b^tm2wtsi^* and carry N-terminal and C-terminal duplications of exons of *Lrp1b*, respectively. Targeting vectors were obtained from the MICER collection [16]. *Lrp1b^tm1wtsi^* mice carry an internal duplication of exons 6–8 of *Lrp1b* while mice with the *Lrp1b^tm2wtsi^* allele carry an internal duplication of exon 69 of Lrp1b (based on the Ensembl predicted gene structure). Both alleles are predicted to cause frameshift mutations.

10 µg of the linearized targeting vectors were electroporated into AB2.2 embryonic stem (ES) cells (from mouse strain 129S5/SvEvBrd) [17]. The ES cells were cultured on a lethally irradiated SNL76/7 feeder layer and picked into 96-well plates after 7 days of drug selection in G418 (180 µg/ml) [18]. To check for homologous recombination, genomic DNA was analyzed by Southern blotting. For the *Lrp1b^tm1wtsi^* allele, a 359 bp 3′ external probe was used (generated by PCR from AB2.2 genomic DNA, using the primers: forward, 5′- AAA AAA TCT TCC TTG AAG GCT CTT GTA AG GTC -3′ and reverse, 5′- ATG CAT ATG GAA TGC CAG GGG GAT GTT CAC AC -3′) and hybridized with *EcoR*V-digested DNA to identify restriction fragments of 18.2 kb for the wild-type and a 11.3 kb for the targeted allele. For the *Lrp1b^tm2wtsi^* allele, a 500 bp external probe was used (generated by PCR from AB2.2 genomic DNA, using the primers: forward, 5′-GAA AGT GAT CAA ATG AAC ATA TTC AAA TCC TTC-3′ and reverse, 5′-CTT GAT CAC AGC TTT CTC TCA ATG GAC TTT AC-3′) on *Bam*HI-digested DNA to identify a 10 kb wild-type and a 20 kb targeted allele.

Correctly targeted ES cell clones were injected into C57BL/6J blastocysts and germline transmission of the targeted (mutant) allele was demonstrated by Southern blot analysis of tail DNA. Mice were maintained on a mixed 129/C57 background and husbandry was in compliance with Home Office regulations (United Kingdom). All animal work was conducted according to the relevant national and international guidelines and in accordance with the recommendations of the Weatherall report, “The use of non-human primates in research” (no primates were used in this study). Animal experiments conducted in Dallas were also reviewed and approved by the Institutional Committee on Animal Use and Care (IACUC) at UT Southwestern Medical Center.

### Isolation and in vitro culture of mouse blastocysts

6–8 week old male and female heterozygous *Lrp1b* mice were intercrossed (with each mouse carrying a different *Lrp1b* allele, such that homozygote embryos could be detected as those carrying both the *Lrp1b^tm1wtsi^* and *Lrp1b^tm2wtsi^* alleles). The females were inspected twice daily for signs of a plug (which was taken as embryonic day 0.5), and three days later the females were sacrificed and their uteri collected and flushed to harvest the blastocysts (embryonic day 3.5). The blastocysts were then cultured in Dulbecco's modified Eagle medium (Invitrogen Ltd, Paisley, UK) supplemented with 10% fetal calf serum, 1 mM L-glutamine, 50 units penicillin/100 µg streptomycin per mL, 1% non-essential amino acids, 0.1 mM β-mercaptoethanol and overlaid with mineral oil in a humidified incubator containing 5% CO_2_ at 37°C for up to 1 week. After culture, each embryo was placed in 20 µL lysis buffer consisting of 50 mM KCl, 10 mM Tris-HCl (pH 8.3), 2.5 mM MgCl_2_, 0.1 mg/mL gelatin, 0.45% Tween-20, 0.45% NP-40 and 1 mg/mL proteinase K. The lysis was carried out at 55°C for 5 hr, followed by 95°C for 15 min. The lysate was then used to perform three separate PCR reactions: to detect the *Lrp1b^tm1wtsi^* allele (forward: 5′-AAA CCG CCT CTC CCC GCG CGT TGG C-3′ and reverse: 5′-CTA TAA GCC AAT CTA ATA AAT TCC CAT CTC TCT-3′), the *Lrp1b^tm2wtsi^* allele (forward: 5′-TGT TTT CAG ACT AGA TAG GCA TTG GGT CTA TA-3′ and reverse: 5′-GCG CCC AAT ACG CAA ACC GCC TCT CCC CG-3′) and an unrelated allele for quality control of the lysate (forward: 5′-GAA GAT GGC TTA GTC GGC CAT CAT TGG GAA GA-3′ and reverse: 5′-GAT GAA TAC ACT GGG TGT GAA ACA CAG CTA CC-3′). The PCR was performed in 50 µL reactions using 45 µL of Platinum PCR Supermix (Invitrogen) and 100 ng of each primer pair with the following PCR cycle profile: 1 cycle at 94°C for 2 min followed by 30 cycles at 94°C for 30 sec, 55°C for 1 min (or 65°C for the *Lrp1b^tm2wtsi^* allele primer pair), and 72°C for 30 sec with a final cycle of 72°C for 10 min. The resulting PCR products were visualized on an ethidium bromide-stained 2% agarose gel.

### Lrp4 *in vitro* assays and Western Blotting

Subconfluent HEK293T cells were grown in 10% FCS/DMEM High Glucose (Cellgro Mediatech Inc.). On day 1, pcDNA3.1 constructs expressing either the extracellular domain or full length murine Lrp4 were transfected into the cells using FuGene6 (Roche Laboratories) according to the manufacturer's protocol. Briefly, 6 µg DNA and 2 uL FuGene6 reagent were suspended (1∶3 ratio of FuGene6∶DNA) in a volume of 600 µL of serum and incubated for 30 min at room temperature before addition to the cell culture dish. The cells were incubated overnight in 10% serum and then switched to serum free DMEM High Glucose/0.2% bovine serum albumin for two days. Cells were collected, washed three times in ice-cold PBS and lysed in 1% Triton-X lysis buffer (50 mM Tris pH 7.4, 150 mM NaCl, 1 mM MgCl_2_, 1 mM CaCl_2_, 1% Triton X-100, with an EDTA-free protease inhibitor cocktail (Complete Mini EDTA-free Protease Inhibitor Cocktail, Roche Laboratories).

The supernatants were centrifuged at 4,000 rpm for 15 min to remove detached or dead cells. Media was then concentrated using 100 kDa size exclusion spin concentrators from Millipore (Amicon Ultra Centrifuge Device 100.000 MWCO). 50 µg cellular extract and 50 µL concentrated media (concentration ∼200∶1) were run on 4–15% gradient gels and subsequently analyzed by immunoblotting for expression of both the intracellular and extracellular domains of Lrp1b and Lrp4. All antibodies were used at a 1∶1,000 dilution in 5% milk/PBS-Tween, and were generated as described below. The bands were visualised by chemiluminescence (Thermo Scientific Pierce ECL Western Blotting) according to the manufacturer's instructions.

Accumulation of ICD was verified with γ-secretase inhibitor DAPT (10 mM, Sigma Aldrich). Treatment was initiated 24 hrs after transfection of the Lrp4 full length construct (murine, pcDNA3.1 vector) for 16 hrs overnight. Cells were then lysed in 1% Triton-X buffer as described previously and 20 µg per lane subjected to Western blotting.

### Antibody generation

The intracellular domain (ICD) specific rabbit polyclonal antibodies for Lrp1b [10] and Lrp4 [15] have been described previously. Extracellular domain (ECD) specific rabbit polyclonal antibodies for Lrp1b and Lrp4 were generated by fusing the murine full ligand binding domain (LBD 2 in case of Lrp1b) to maltose binding protein (MBP) followed by bacterial expression of the recombinant fusion protein. Briefly, DH5α *Ε. coli* cells were transformed and induced with IPTG (0.5 µM) overnight for 16 hrs. Bacteria were then centrifuged and exposed to osmotic shock to release the correctly folded fusion protein from the periplasmic space. Proteins were then column-purified and injected subcutaneously into 3 months old rabbits. The immunization was repeated every four weeks until high titer antibodies were obtained.

### TOP-Flash Assay

HEK-293 cells were plated at 400 000 cells/well in 6-well plates and grown to 50–80% confluency in 10% FBS/DMEM. Cells were transfected using the TOP-Flash reporter assay system [11] with the indicated expression plasmids for Wnt1, Dkk1, Lrp4 ECD, Lrp5 and Lrp6 in pcDNA3.1 backbones (0.5 µg/construct, 2.5 µg total). To account for the different numbers of transfected plasmids, empty pcDNA 3.1 plasmid was added to a total of 2.5 µg DNA/well. Transfections were performed with FuGene6 using the manufacturer's protocol. Cells were lysed 48 hrs after transfection and lysates were assayed for firefly and renilla luciferase activities using the Dual Luciferase Reporter Assay System (Promega), according to the manufacturer's protocol. All transfections and measurements were performed in triplicate.

## Results

### Absence of Lrp1b in Mice Results in Early Embryonic Lethality

**Figure 1:**
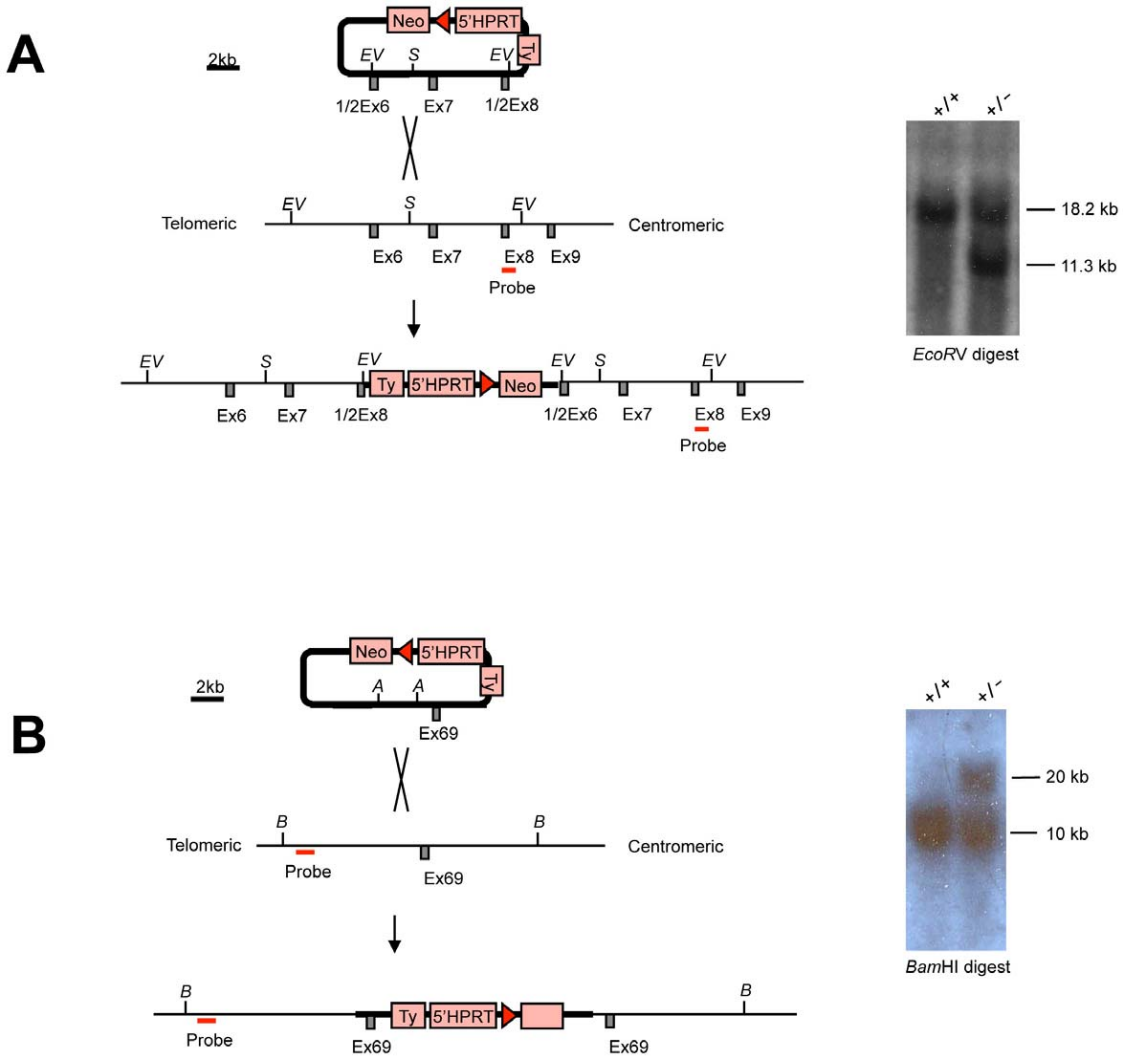
Generation of *Lrp1b* null alleles. (**A**) Duplication of N-terminal exons 6-8 to generate the *Lrp1b^tm1wtsi^* allele and Southern blot hybridization after *EcoR*V digestion of embryonic stem cell genomic DNA to verify targeting of the allele. (**B**) Duplication of C-terminal exon 69 to generate the *Lrp1b^tm2wtsi^* allele and Southern blot hybridization after *BamH*I digestion of embryonic stem cell genomic DNA to verify targeting of the allele. *A*, *Afl*III; *B*, *BamH*I; *EV*, *EcoR*V; *S*, *Swa*I.

We generated two different *Lrp1b* null alleles – the first targeting the N-terminus with duplication of exons 6–8 (*Lrp1b^tm1wtsi^* mice), and the second targeting the C-terminus with duplication of exon 69 (*Lrp1b^tm2wtsi^* mice); both mutations result in premature termination through frameshift (Figure 1). ES cells carrying these alleles were used to generate chimaeras, which transmitted the targeted alleles to their progeny. Heterozygous mice (*Lrp1b^tm1wtsi/+^* and *Lrp1b^tm2wtsi/+^*) were healthy at birth and both males and females were fertile. However, no homozygous mice of either allele were observed at weaning (Table 1). To analyze this in more detail, we focused on just one of the alleles. Using the *Lrp1b^tm2wtsi^* allele, a total of 146 mice were genotyped at weaning (4 weeks old). No homozygous *Lrp1b^tm2wtsi^* mice were detected, suggesting that homozygous *Lrp1b* mice were not viable (Table 1). We then isolated embryos at E8.5 and E10.5 for genotyping by Southern hybridization but did not find any homozygous *Lrp1b* embryos at these timepoints indicating that *Lrp1b* disruption caused early embryonic lethality (Table 1).

**Table 1:** Genotyping of Lrp1b heterozygous intercrosses.

	LRP1b		
Age	+/+	+/−	−/−	TOTAL	Fisher's exact test
4 weeks	45	101	0	146	p<0.0001
E10.5	5	22	0	27	p<0.01
E8.5	4	15	0	19	p<0.05
E3.5	11	11	3	25	NS

Genotyping data at 4 wks, E8.5 and E10.5 was performed on genomic DNA from intercrosses of *Lrp1b^tm2wtsi^* mice, whereas genotyping of the E3.5s was performed on genomic DNA from intercrosses of *Lrp1b^tm1wtsi^* with *Lrp1b^tm2wtsi^* mice. Experimental data was statistically analyzed using Fisher's exact test. NS, not significant.

### Lrp1b-deficient Blastocysts Are Viable

**Figure 2:**
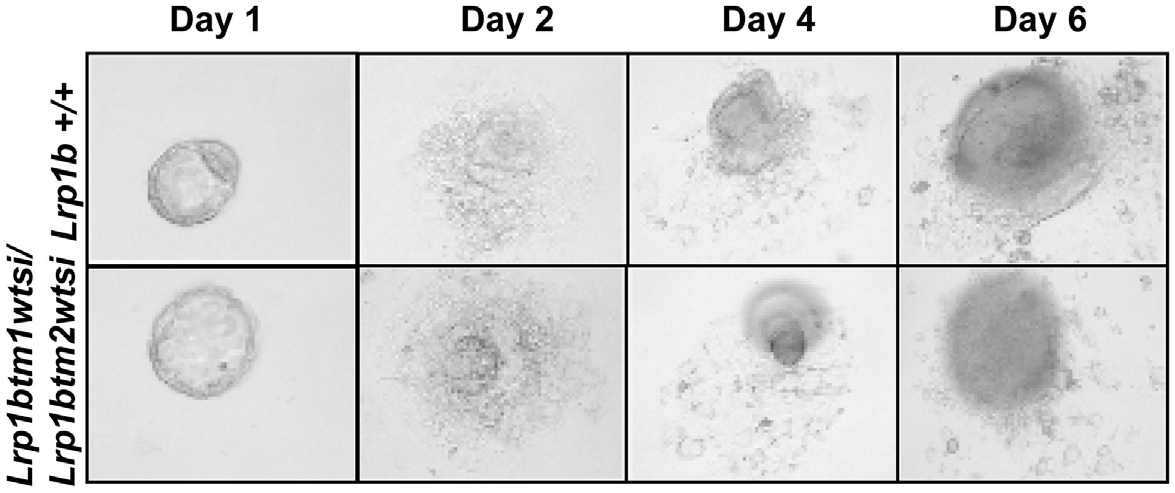
Blastocyst outgrowth assay. Time course of *Lrp1b* wildtype (*Lrp1b^+/+^*) compared to *Lrp1b* knockout (*Lrp1b^tm1wtsi/tm2wtsi^*) trophoblast explant growth, showing expansion of inner cell mass and trophoblast formation. Images were taken on days 1, 2, 4, and 6.

Pre-implantation embryos do not provide sufficient material for Southern analysis and PCR genotyping cannot distinguish homozygous embryos for each of the mutant alleles individually from heterozygous embryos. Therefore in order to narrow the timepoint when embryos in which *Lrp1b* had been disrupted die we intercrossed *Lrp1b^tm1wtsi^* and *Lrp1b^tm2wtsi^* mice, flushed blastocysts at E3.5 and cultured these to form blastocyst outgrowths. In total, 25 blastocyst outgrowths were analyzed by PCR for the presence of both mutant alleles which would indicate homozygous null embryos. Three of these were genotyped as homozygotes (p>0.1). As shown in Figure 2 these blastocysts showed normal morphology with a time-and size-appropriate expansion of the inner cell mass and outgrowth of trophoblast structures. This result suggests that loss of *Lrp1b* does not result in a cell lethal phenotype.

### Extracellular Domains Are Expressed by Truncated Lrp1b and Lrp4 Alleles

**Figure 3:**
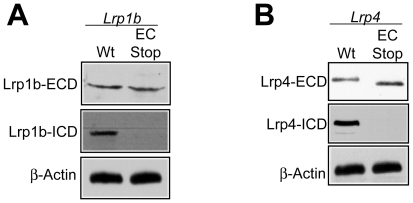
Expression of Lrp1b and Lrp4 ECDs. Whole brain lysates (50 µg) from (**A**) Lrp1b and (**B**) Lrp4 truncation mutants were analyzed for expression of the ECD. For the *Lrp1b* truncation (“Lrp1b EC Stop”), the ECD is expressed at approximately the same size as the full-length receptor (“Wt”) due to the negligible reduction in predicted protein mass. However, as expected the ICD epitope is only present in wild-type tissues. By contrast, in the *Lrp4* truncation (“Lrp4 EC Stop”), there is a significant shift in size of the ECD protein band compared to full-length receptor. As for Lrp1b, no ICD is detected in the truncated Lrp4 strain. β-Actin was used as a loading control.

The expression of the extracellular domains (ECDs) in the previously reported knockout models of *Lrp1b* [10] and *Lrp4* [11], [13] was predicted but never confirmed. To confirm the expression of Lrp1b and Lrp4 ECDs, we utilized whole brain lysates and antibodies against the extracellular and intracellular ligand binding domains. For Lrp1b, only a slight size difference was detectable between the protein products from the wild-type and the Lrp1b truncation allele (Figure 3A). However, the intracellular domain epitope was only detectable in the wild type. For Lrp4, the size difference confirmed the expression of the predicted 180 kDa Lrp4-ECD in the absence of an ICD (Figure 3B). Expression levels of the truncated ECDs were equivalent to wild type full-length protein. We thus confirmed that the extracellular domains are expressed normally and are stable in both mutant strains.

### Lrp4 Undergoes Regulated Intramembranous Processing *In Vitro*

**Figure 4:**
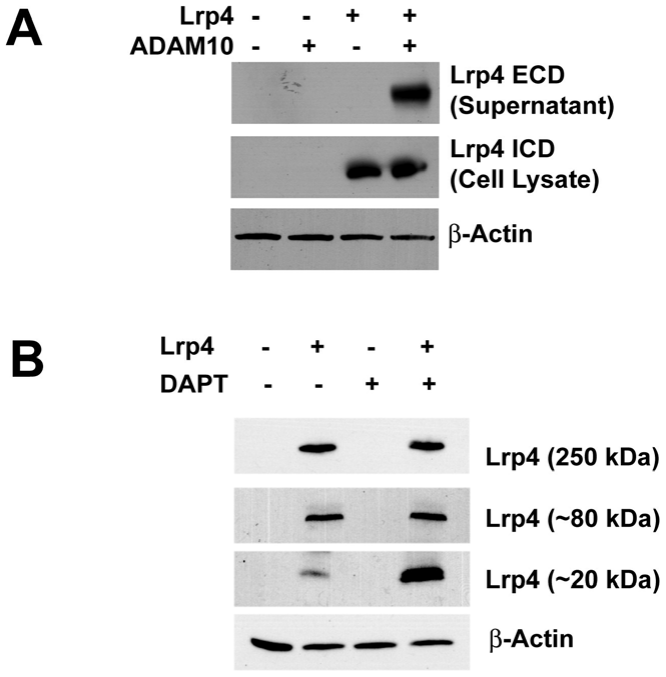
Lrp4 undergoes regulated intramembraneous processing. (A) Lrp4 ECD release is induced by ADAM10 *in vitro*. 50 µL of concentrated supernatant and 50 µg of cell lysate were analyzed with a polyclonal antibody detecting either the Lrp4 extracellular domain (Lrp4 ECD) domain (supernatant) or the Lrp4 intracellular domain (cellular extracts). The extracellular domain is present in the supernatant after transfection with Lrp4 and co-transfection with the metalloproteinase Adam10 (lane 4), but not in the absence of Adam10 (lane 3). Immunoblotting for β-actin was used to demonstrate equal loading. **(B) Lrp4 ICD is cleaved by γ-secretase.** Lrp4 expression in 293T cells reveals bands at 20, 75, and 250 kDa (lanes 2 and 4). The protein levels of 250 and 75 kDa species are independent of DAPT (i.e. γ-secretase inhibitor) treatment. By contrast, the membrane bound ICD at 20 kDa accumulates in the presence of DAPT. No protein products were detected in the untransfected lanes (1 and 3). β-Actin was detected to demonstrate equal loading.

It has previously been reported that the ECD of Lrp1b is shed into the extracellular space in an in vitro model and the ICD is released by γ-secretase activity [21]. To investigate whether Lrp4 is similarly processed and the extracellular domain shed into the extracellular space, the supernatants of Lrp4-transfected cells were analyzed by immunoblotting using an antibody against the ECD of Lrp4 (Figure 4A). Cell lysates were used to verify transfection efficiency using the Lrp4 ICD antibody. No shed ECD was detected in the supernatant from untransfected cells (lane 1) or cells that had been transfected with either ADAM10 (lane 2) or the full length Lrp4 construct (lane 3) alone. When the Adam10 metalloproteinase was co-transfected together with Lrp4 to facilitate cleavage of the extracellular domain, Lrp4-ECD was released from the cell and became readily detectable in the culture supernatant (lane 4).

Transfection of Lrp4 reveals different protein products at ∼20 kDa, 75 kDa and 250 kDa (Figure 4B, lanes 2 and 4); while the 250 kDa band represents full length Lrp4, the two smaller bands appear to be processing products of the receptor. No bands were detected in the untransfected conditions (Figure 4B, lanes 1 and 3). In analogy to other members of the LDL receptor gene family, the processing of Lrp4 includes extracellular domain cleavage by metalloproteases and a release of the ICD by γ-secretase activity. Inhibition of γ-secretase by DAPT correlates with accumulation of the ∼20 kDa band.

### The Lrp4 ECD Inhibitis Canonical Wnt Signaling

**Figure 5:**
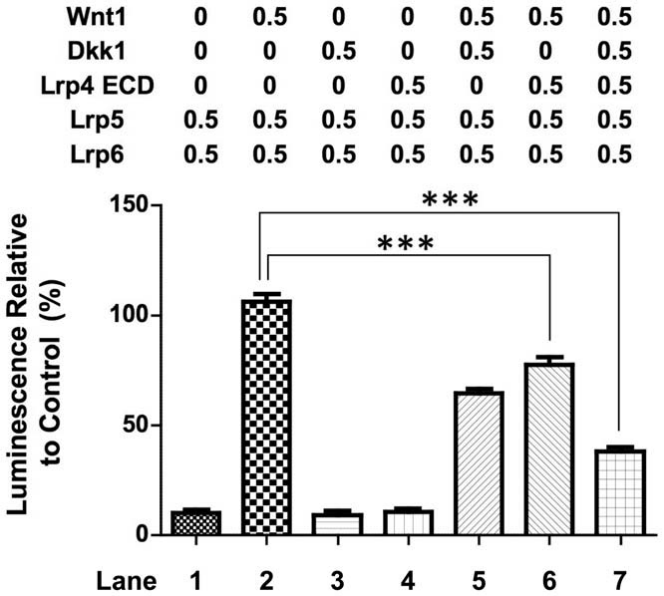
Lrp4 ECD Inhibits Wnt signaling *in vitro*. HEK-293 cells were transfected using the TOP-Flash reporter system in the presence of the indicated plasmids (0.5 µg/construct as indicated, where required empty pcDNA3.1 plasmid DNA was added to bring the DNA concentration up to a total amount of 2.5 µg plasmid DNA/condition). Dkk1 and Lrp4 independently inhibit Wnt signaling (lane 5 and 6). Inhibition of Wnt1 induced reporter activation by co-transfection of Lrp4 ECD and Dkk-1 is synergistic (lane 7). Lrp5 and Lrp6 are co-receptors of the frizzled complex and required for Wnt1 mediated activation, however, HEK-293T cells do not express Lrp5 or 6 endogenously and thus need to be co-transfected with the respective plasmids [11].

Lrp4 has been reported to be a negative regulator of Wnt signaling. To investigate whether the ECD can mediate this inhibition on its own, we used a TOP-Flash assay system and measured β-catenin dependent promoter activity *in vitro*. Wnt1 was used to activate signaling at the extracellular level. Dickkopf-1 (Dkk1) has been reported to be a negative regulator of Wnt signaling [19] and an Lrp4 binding partner [20]. As expected, Lrp4 and Dkk1 do not repress Wnt reporter activity in the absence of Wnt1 (Figure 5, lanes 3 and 4). However, in the presence of Wnt1, both Dkk1 (Figure 5, lane 5) and Lrp4 (Figure 5, lane 6) can independently decrease Wnt signaling significantly. In combination, Dkk1 and Lrp4 synergistically increase Wnt1 signal inhibition (Figure 5, lane 7).

## Discussion

In this study, we have presented evidence for an essential role of *Lrp1b* during embryonic development. From two different *Lrp1b* null alleles, no viable offspring or embryos were obtained. Although blastocyst outgrowths appeared normal, we were unable to identify viable homozygous *Lrp1b* mutant embryos at or beyond E8.5, suggesting that loss of *Lrp1b* causes early embryonic lethality and underscoring the importance of this gene for embryonic development. We have previously reported that mice carrying a truncated form of *Lrp1b* exclusively expressing a secreted ECD, are born at normal Mendelian ratios and are phenotypically essentially normal. In this earlier study, we had used insertion of a ‘neomycin’ cassette to replace the transmembrane domain at exon 88 of *Lrp1b*, resulting in the predicted truncation of the receptor and the secretion of a fully folded and functionally apparently intact ECD [10]. Under physiological conditions, *LRP1b* is anchored through its transmembrane domain in the cell membrane where it can undergo regulated intramembrane proteolysis (RIP) [21]. The ECD of *LRP1b* is cleaved by several metalloproteinases, including ADAM17 and other members of the ADAM family, in the initial step of receptor processing and leads to shedding into the extracellular space where its function has not yet been determined [21]. Subsequently, γ-secretase activity mediates the release the intracellular domain from the membrane. LRP1 and other members of the LDL receptor gene family are known to bind a wide variety of ligands, including growth factors, membrane receptors, the amyloid precursor protein, bacterial toxins, and other proteins [14]. Given their structural similarities, *LRP1b* is also likely to bind a comparable spectrum of ligands. In fact, the amyloid precursor protein [22], Pseudomonas exotoxin A [23] and some other ligands have been reported to also bind to the ECD of LRP1b. Our gene targeting study to disrupt *Lrp1b* by duplicating internal exons of the gene suggests that the ECD can function independently from the membrane anchored receptor to regulate critical developmental processes required for embryonic viability. The shedding of the ECD into the extracellular space might therefore serve as a soluble ligand scavenger. This event presumably preserves a critical signaling threshold at an early stage of embryonic development.

For other members of the LDL receptor gene family, it has been demonstrated that the cleavage of the extracellular domain can occur in the native receptor [24]. Interestingly, we have found a comparable rescue of a severe perinatally lethal phenotype by a truncated form of *Lrp4*, where only the ECD remains expressed [11], [13]. Here, we confirmed Lrp4 ECD expression in this mutant mouse strain and present *in vitro* evidence that Lrp4 undergoes regulated intramembraneous processing (RIP) by cleavage and shedding of the ECD by metalloproteases and ICD release after γ-secretase cleavage. Both steps have important physiological functions in other LDL gene family members including signal modulation and transcriptional inhibition.

Furthermore, our *in vitro* results suggest that Lrp4 ECD can negatively modulate Wnt signaling. Whether this happens through cooperation with inhibitory ligands or scavenging of activating ligands extracellularly remains to be determined. It also remains presently unclear whether shedding occurs *in vivo* and on which physiological processes this may impact. However, anchorage-independent modulation of extracellular conditions seems to play a crucial role in preserving a threshold for proper cellular signal input. No specific signaling mechanisms, which are modulated by Lrp1b are currently known. This hypothesis thus requires further confirmation once such pathways have been identified.

Deletion of *Lrp4* causes perinatal death due to an inability to form neuromuscular junctions and subsequent respiratory failure [12]. This phenotype is mitigated in the truncated *Lrp4* receptor expressing only the ECD, allowing the animal to breathe and move, despite general muscular weakness and hypotrophy. Another prominent phenotype, involving abnormal distal limb development, appears to be identical in the null and hypomorph [11], [12].

There are several reports of *LRP1b* being deleted or epigenetically silenced in a variety of human tumors [3], [4], [5], [7]. The exact mechanistic role of *LRP1b* in tumor suppression and development has remained elusive. The previously reported functional insights into tumor suppression at the molecular level overlap with its close relative *LRP1*. They include the regulation of uPA, uPAR and PDGF receptor tyrosine kinase [8], [25]. However, the lack of mutations in *LRP1* [2] indicate important functions that have diverged from those of *LRP1b*. These differences could be attributed to the distinct selective pressure on the *LRP1b* gene in the process of tumor development. It is thus possible that the same unknown mechanisms that are regulated by the Lrp1b ECD are involved in tumorigenesis as well as development.

While the release of the intracellular domain and its effect on inflammatory signaling and proliferation has been described for both *LRP1b* [21] and *LRP1* [26], no such independent function has been described for the isolated ECDs of either receptor. Our data, obtained from two distinct mouse models suggest that the ECD of *Lrp1b* can function to some extent to maintain signaling homeostasis even in the absence of membrane integration. In analogy to LRP1, this might occur through binding of soluble ligands in the extracellular space [27].

**Figure 6:**
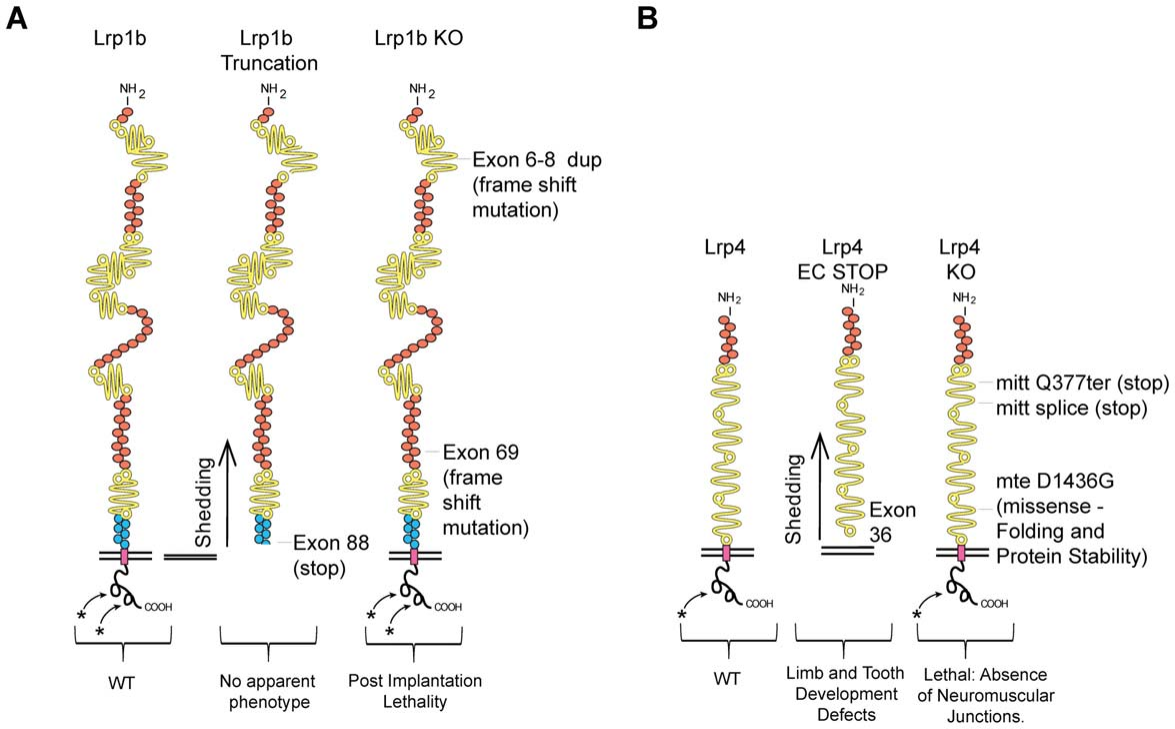
Summary of known mutations and their respective phenotypes. The known mutations in murine models for (**a**) *Lrp1b* and (**b**) *Lrp4* are shown. The presence of the extracellular domain (ECD) rescues the lethality caused by the complete functional null mutation.

In summary, we have reported an essential role for *Lrp1b* in embryonic development and propose a novel role for *Lrp1b* and *Lrp4* as signal modulators through ligand scavenging (Figure 6). Further elucidation of the molecular functions of the LRP1b and LRP4 ECDs has the potential to provide novel and functionally significant insights into the role of *LRP1b* in embryogenesis and cancer.
